# Post-haemorrhagic hydrocephalus is associated with poorer surgical and neurodevelopmental sequelae than other causes of infant hydrocephalus

**DOI:** 10.1007/s00381-021-05226-4

**Published:** 2021-06-19

**Authors:** Malak Mohamed, Saniya Mediratta, Aswin Chari, Cristine Sortica da Costa, Greg James, William Dawes, Kristian Aquilina

**Affiliations:** 1 UCL Great Ormond Street Institute of Child Health, University College London, London, UK; 2 Leeds School of Medicine, University of Leeds, Leeds, UK; 3 Division of Neurosurgery, Department of Clinical Neurosciences, Addenbrooke’s Hospital & University of Cambridge, Cambridge, UK; 4 Department of Neurosurgery, Great Ormond Street Hospital, London, UK; 5 Department of Neonatology, Great Ormond Street Hospital, London, UK; 6 Department of Neurosurgery, Alder Hey Children’s Hospital, Liverpool, UK

**Keywords:** Intraventricular haemorrhage, Hydrocephalus, Neonates, Ventriculoperitoneal shunts, Neurodevelopmental outcome

## Abstract

**Purpose:**

This retrospective cohort study aimed to investigate the surgical and neurodevelopmental outcomes (NDO) of infant hydrocephalus. We also sought to determine whether these outcomes are disproportionately poorer in post-haemorrhagic hydrocephalus (PHH) compared to other causes of infant hydrocephalus.

**Methods:**

A review of all infants with hydrocephalus who had ventriculoperitoneal (VP) shunts inserted at Great Ormond Street Hospital (GOSH) from 2008 to 2018 was performed. Demographic, surgical, neurodevelopmental, and other clinical data extracted from electronic patient notes were analysed by aetiology. Shunt survival, NDO, cerebral palsy (CP), epilepsy, speech delay, education, behavioural disorders, endocrine dysfunction, and mortality were evaluated.

**Results:**

A total of 323 infants with median gestational age of 37.0 (23.29–42.14) weeks and birthweight of 2640 g (525–4684 g) were evaluated. PHH was the most common aetiology (31.9%) and was associated with significantly higher 5-year shunt revision rates, revisions beyond a year, and median number of revisions than congenital or “other” hydrocephalus (all *p* < 0.02). Cox regression demonstrated poorest shunt survival in PHH, related to gestational age at birth and corrected age at shunt insertion. PHH also had the highest rate of severe disabilities, increasing with age to 65.0% at 10 years, as well as the highest CP rate; only genetic hydrocephalus had significantly higher endocrine dysfunction (*p* = 0.01) and mortality rates (*p* = 0.04).

**Conclusions:**

Infants with PHH have poorer surgical and NDO compared to all other aetiologies, except genetic hydrocephalus. Research into measures of reducing neurodisability following PHH is urgently required. Long-term follow-up is essential to optimise support and outcomes.

**Supplementary information:**

The online version contains supplementary material available at 10.1007/s00381-021-05226-4.

## Introduction

Infant hydrocephalus is often associated with developmental delay and multiple comorbidities [1, 2]. Both surgical and neurodevelopmental outcomes (NDO) have been shown to vary depending on the aetiology of hydrocephalus, with mixed findings in the literature in terms of ventriculoperitoneal (VP) shunt failure rates [3–7]. The importance of measuring NDO, rather than surgical outcomes only, in paediatric hydrocephalus, has been emphasised [8, 9]. As most studies are retrospective and not constrained to infant hydrocephalus alone, separating the effects of the hydrocephalus from the underlying pathology can be difficult. Additionally, the variation between studies on the influence of hydrocephalus aetiology on NDO is a possible reflection of the heterogeneity of the underlying disorders [5].

Post-haemorrhagic hydrocephalus (PHH), which occurs following intraventricular haemorrhage (IVH) of prematurity, is hypothesised to be associated with poorer outcomes than other aetiologies. In addition to the raised intracranial pressure (ICP) and distortion of developing neural tracts caused by hydrocephalus, pro-inflammatory cytokines and free radicals from iron due to haemoglobin breakdown cause toxicity and brain injury [10, 11]. Prematurity is also an independent risk factor for poor NDO and has been associated with high rates of cerebral palsy (CP), epilepsy, and visual impairment [12, 13]. Premature neonates with VP shunts have higher rates of shunt infection and obstruction secondary to immature immune systems and high cerebrospinal fluid (CSF) protein levels and red blood cells [14–16].

This retrospective cohort study aims to evaluate the surgical and NDO of infant hydrocephalus and to investigate whether PHH has disproportionately poorer outcomes compared to other hydrocephalus aetiologies. This will help provide prognostic information for clinicians and families and will highlight any areas for improvement of patient outcomes.

## Methods

### Study design

This retrospective cohort study is reported according to STROBE guidelines (checklist available in Supplementary Information 1) and was registered as a service evaluation with the Great Ormond Street Hospital (GOSH) Clinical Audit Department [17].

### Inclusion criteria

Any primary VP shunt insertion at GOSH within the first year of life, between January 2008 and January 2018, was eligible for inclusion. There were no exclusion criteria. Patients were identified using a prospectively collected neurosurgical operative database.

### Data collection

Demographic, surgical, neurodevelopmental, and other clinical data were extracted from the electronic health record system. Data for all patients were collected until death, loss to follow-up, or the end of the data collection period; the last date of follow-up was recorded for each patient to allow analysis which accounts for follow-up time.

The aetiology of hydrocephalus was classified into PHH (infants born < 37 weeks with IVH diagnosed on cranial ultrasound), neural tube defects (NTDs, including myelomeningoceles and encephaloceles), congenital (including aqueduct stenosis, brain cysts, and other complex congenital brain malformations), genetic (X-linked hydrocephalus, craniofacial abnormalities, and other suspected genetic syndromes), or “other” hydrocephalus. The “other” group combined miscellaneous aetiologies with low patient numbers and included infection, tumours, trauma, term IVH, and unknown causes.

Surgical variables included the use of a temporising neurosurgical procedure (TNP) and the method used, corrected age at VP shunt insertion, type of shunt, shunt complications, and number of, and time to any, shunt revisions. Where an infected shunt was replaced by a drain prior to re-insertion, removal and re-insertion were counted as one revision, and the revision date recorded was the removal date of the infected shunt. NDO scores were assigned by one author (MM), using the scale developed by Resch et al. (Table 1), using data from developmental clinic notes, neurosurgical reviews, and paediatric follow-up [18]. NDO was reported at 1, 2, 5, and 10 years, corrected for prematurity. Only infants who had reached the age of each assessment were scored for that time point.

**Table 1:** Neurodevelopmental outcome scale developed by Resch et al. [18]

Outcome score	Description of neurodevelopment
0	Normal for corrected age, no neurological abnormalities
1	Slight developmental delay, mild neurological abnormalities including mild hypertonia/hypotonia, abnormal coordination, isolated hyperreflexia, nystagmus, or strabismus
2	Disabilities like mono-, di-, and hemiparesis, mild visual or hearing impairment, well-controlled seizures, and/or mild learning disability
3	Severe disabilities like tri- or quadriparesis, blindness, deafness, poorly controlled seizures, and/or severe learning disability

Only cerebral visual impairments and sensorineural hearing losses attributable to hydrocephalus were included in NDO scores. Seizure control was defined by the continuation of seizures while on antiepileptic medication or post-epilepsy surgery; febrile seizures were excluded

In addition to NDO, clinical outcomes including CP, epilepsy, speech delay, type of education for children who had reached school age (mainstream, supported, or special schooling), behavioural disorders, endocrine dysfunction, and mortality (including age and cause of death) were recorded. Children who died before any clinical outcomes could be excluded were removed from the analysis of that clinical outcome, and infants lost to follow-up were excluded from mortality analysis. For cases where outcomes were likely influenced by concomitant pathology, a sensitivity analysis, which excluded those infants, was conducted.

### Statistical analysis

A quantitative analysis was performed using SPSS version 26.0. Categorical data were summarised as frequencies and percentages; as numerical variables were non-parametric, median and range were used. Chi-squared tests compared categorical variables between aetiology groups where expected frequency counts were ≥ 5 and Fisher’s exact test where counts were < 5. Ordinal data were analysed using the chi-squared test for trend and numerical data using the Kruskal–Wallis test. Where significant differences (*p* < 0.05) existed between aetiology groups, pairwise comparisons were adjusted for multiple testing using Bonferroni’s correction (*p* < 0.0125).

Shunt survival across aetiology groups was evaluated by Cox regression, and the effect of aetiology on NDO and other clinical outcomes was explored with ordinal, binary, and multinomial logistic regression. In multivariate regression, for each outcome variable studied, a set of potential confounders identified from the literature was selected for a priori inclusion in the final model, and the remaining potential predictors were then entered into the model using Backward (Wald) elimination (threshold of significance *p* < 0.05). PHH was the reference group for all regression analyses.

## Results

### Patient demographics

A total of 323 infants met the study’s inclusion criteria. Infants lost to follow-up, either due to death (*n* = 30) or discharge from follow-up (*n* = 21), were included in the study until death or discharge only. Median follow-up was 6.46 years (0.02–13.22) for all included infants. The most common aetiologies of hydrocephalus were PHH of prematurity (*n* = 103, 31.9%), myelomeningocele (*n* = 36, 11.1%), and term IVH (*n* = 29, 9.0%) (Table 2).

**Table 2:** Aetiologies of hydrocephalus

	Frequency, n (%)
Post-haemorrhagic hydrocephalus of prematurity	103 (31.9%)
Neural tube defects Myelomeningocele Encephalocele	40 (12.4%) 36 (11.1%) 4 (1.2%)
Genetic hydrocephalus Craniofacial abnormality X-linked hydrocephalus Other genetic syndromes	45 (13.9%) 13 (4.0%) 6 (1.9%) 26 (8.0%)
Congenital hydrocephalus Aqueduct stenosis Brain cyst Blake’s pouch cyst: Interhemispheric arachnoid cyst Porencephalic cyst Posterior fossa arachnoid cyst Other congenital brain malformations	62 (19.2%) 17 (5.3%) 25 (7.7%) 4 (1.2%) 4 (1.2%) 4 (1.2%) 13 (4.0%) 20 (6.2%)
Others Term intraventricular haemorrhage Infection Brain tumour Haematoma Trauma Unknown	73 (22.6%) 29 (9.0%) 16 (5.0%) 14 (4.3%) 2 (0.6%) 2 (0.6%) 10 (3.1%)

Other genetic syndromes combined all infants who had a genetic diagnosis of hydrocephalus, or for whom the genetics team highly suspected a genetic aetiology, but the precise syndrome had yet to be identified at the time of the study. Examples included Joubert syndrome, CHARGE syndrome, Walker-Warburg syndrome, Aicardi syndrome, and Galloway-Mowat syndrome. Other congenital brain malformations were complex congenital malformations with no obvious genetic cause, including holoprosencephaly, hydranencephaly, hemimegalencephaly, schizencephaly, septo-optic dysplasia, and Dandy Walker malformation. Term IVH was treated as a separate entity to PHH of prematurity. The pathophysiology of the brain injury in these neonates is different from that in preterm IVH; there is no germinal matrix injury, no periventricular infarction, and brain development is at a later stage. Management was also different; if there was acute hydrocephalus, infants who suffered term IVH underwent external ventricular drainage rather than insertion of a VSGS or an access device. When necessary, they underwent VP shunt insertion as soon as the intraventricular blood resolved

The baseline characteristics of the cohort are described by aetiology (Table 3). Males were more frequent in all aetiology groups, with no significant differences in sex between groups (*p* = 0.54). Significantly, more infants were preterm in the PHH group than all other aetiologies, with a lower median gestational age of 27 weeks (*p* < 0.01) and correspondingly lower birthweights (*p* < 0.01). Most PHH infants had grade IV (54.3%) or grade III (27.2%) IVH. Between 2008 and 2012, intervention on PHH infants was only considered for clinical evidence of raised ICP; between 2013 and 2018, intervention was undertaken earlier, based on a ventricular index at the 97th centile plus 4 mm, according to the Levene chart [19]. Forty-two (40.8%) PHH infants had a TNP prior to VP shunt insertion.

**Table 3:** Patient characteristics of cohort by aetiology

Parameter	Frequency (%)/median (range)						
	Total (*n* = *323*)	PHH (*n* = *103*)	NTDs (*n* = *40*)	Genetic (*n* = *45*)	Congenital (*n* = *62*)	Others (*n* = *73*)	*p* value
Female	137 (42.4%)	37 (35.9%)	17 (42.5%)	22 (48.9%)	27 (43.5%)	34 (46.6%)	0.54
Male	186 (57.6%)	66 (64.1%)	23 (57.5%)	23 (51.1%)	35 (56.5%)	39 (53.4%)	
Gestational age (weeks)	37.00 (23.29–42.14)	27.00 (23.29–35.29)	38.07 (33.00–40.86)*	38.00 (29.57–41.43)*	38.71 (30.00–42.14)*	39.00 (27.29–42.00)*	< 0.01
Preterm	150 (46.4%)	103 (100.0%)	8 (20.0%)*	14 (31.1%)*	18 (29.0%)*	7 (9.6%)*	< 0.01
Birthweight (g)	*n* = *275* 2640 (525–4684)	*n* = *97* 904 (530–2754)	*n* = *36* 2880 (1760–4500)*	*n* = *40* 2937.5 (525–4328)*	*n* = *44* 3210 (1040–4600)*	*n* = *58* 3249 (1060–4684)*	< 0.01
Papile grade:		*n* = *92*					
Grade I		7 (7.6%)					
Grade II		10 (10.9%)					
Grade III		25 (27.2%)					
Grade IV		50 (54.3%)					
TNP˟:	61 (18.9%)	42 (40.8%)	2 (5.0%)*	2 (4.4%)*	2 (3.2%)*	13 (17.8%)*	< 0.01
VSGS	35 (10.8%)	35 (34.0%)	0 (0.0%)*	0 (0.0%)*	0 (0.0%)*	0 (0.0%)*	< 0.01
VAD	13 (4.0%)	8 (7.8%)	0 (0.0%)	1 (2.2%)	2 (3.2%)	2 (2.7%)	0.28
EVD	23 (7.1%)	7 (6.8%)	2 (5.0%)	1 (2.2%)	1 (1.6%)	12 (16.4%)	0.01

*p* values were obtained using the following: chi-squared test: sex, preterm, TNP; Fisher’s exact test: VSGS, VAD, EVD; Kruskal–Wallis test: gestational age, birthweight

*PHH* post-haemorrhagic hydrocephalus, *NTDs* neural tube defects, *TNP* temporising neurosurgical procedure, *VSGS* ventriculosubgaleal shunt, *VAD* ventricular access device, *EVD* external ventricular drain

**˟**Percentages do not add up as some infants had multiple temporising interventions

^*^Parameters that are statistically significant between that aetiology group and PHH

### Surgical outcome

#### Surgical procedures

The median corrected age at VP shunt insertion was 1 day before term-equivalence in the PHH group, which was significantly younger than genetic, congenital, and “other” hydrocephalus (*p* < 0.01). Most infants (90.1%) had fixed pressure valves, with only 13 (4.0%) having programmable valves; the distribution of shunt type was different across groups (*p* = 0.01; Table 4).

**Table 4:** Surgical parameters and outcomes by aetiology

Parameter	Frequency (%)/median (range)						
	Total (*n* = *323*)	PHH (*n* = *103*)	NTDs (*n* = *40*)	Genetic (*n* = *45*)	Congenital (*n* = *62*)	Other (*n* = *73*)	*p* value
Type of VP shunt:							0.01
Fixed pressure	291 (90.1%)	88 (85.4%)	39 (97.5%)	41 (91.1%)	59 (95.2%)	64 (87.7%)	
Valveless	19 (5.9%)	13 (12.6%)	0 (0.0%)	0 (0.0%)*	1 (1.6%)	5 (6.8%)	
Programmable	13 (4.0%)	2 (1.9%)	1 (2.5%)	4 (8.9%)	2 (3.2%)	4 (5.5%)	
Corrected age at VP shunt insertion (days)	17.0 (− 88 to 359)	− 1.0 (− 88 to 238)	1.5 (− 37 to 251)	43 (− 31 to 359)*	28 (− 63 to 324)*	54 (− 18 to 357)*	< 0.01
	*Excluding those with follow-up < 1 year of shunt insertion:*						
	*n* = *302*	*n* = *96*	*n* = *39*	*n* = *38*	*n* = *59*	*n* = *70*	
Revision rate ≤ 1 year	102 (33.8%)	36 (37.5%)	16 (41.0%)	16 (42.1%)	19 (32.2%)	15 (21.4%)	0.11
Number of revisions ≤ 1 year of shunt	0 (0–4)	0 (0–4)	0 (0–3)	0 (0–3)	0 (0–2)	0 (0–3)	0.10
	*Excluding those with follow-up < 5 years of shunt insertion:*						
	*n* =*203*	*n* = *59*	*n* = *25*	*n* = *24*	*n* = *41*	*n* = *54*	
Revision rate > 1 year	51 (25.1%)	24 (40.7%)	4 (16.0%)	7 (29.2%)	7 (17.1%)*	9 (16.7%)*	0.02
Number of revisions > 1 year of shunt	0 (0–5)	0 (0–5)	0 (0–2)	0 (0–4)	0 (0–2)*	0 (0–4)*	0.03
5-year revision rate	95 (46.8%)	37 (62.7%)	10 (40.0%)	13 (54.2%)	16 (39.0%)	19 (35.2%) *	0.03
Total number of revisions < 5 years per patient	0 (0–9)	1 (0–9)	0 (0–4)	1 (0–4)	0 (0–3)*	0 (0–5)*	0.02

As each patient had their shunt in situ for different lengths of time, evaluation of revisions beyond a year of insertion was limited to patients with a minimum of 5 years of follow-up post shunt, and only a 5-year follow-up was evaluated. Revision rates are defined as the proportion of patients who required VP shunt revisions due to shunt complications. p values were obtained using the following: Chi-squared test: revision rate ≤ 1 year, revision rate > 1 year, and 5-year revision rate; Fisher’s exact test: type of VP shunt; Kruskal–Wallis test: corrected age at VP shunt insertion, number of revisions ≤ 1 year of shunt, number of revisions > 1 year of shunt, and total number of revisions < 5 years per patient

*PHH* post-haemorrhagic hydrocephalus, *NTDs* neural tube defects, *VP* ventriculoperitoneal

^*^Parameters that are *statistically significant* between that aetiology group and PHH

#### Shunt revisions

Neither 1-year revision rate nor number of revisions in the first year was different between aetiologies (*p* = 0.11, *p* = 0.10 respectively). However, revision rate > 1 year, number of revisions > 1 year, 5-year revision rate, and total number of revisions at 5 years were significantly different between groups (*p* = 0.02, *p* = 0.03, *p* = 0.03, and *p* = 0.02, respectively), with the rates and medians being highest in the PHH group. Revision rates were higher in preterm PHH than in term IVH, although this did not reach significance (Supplementary Information 2).

#### Time to first shunt revision

**Fig. 1:**
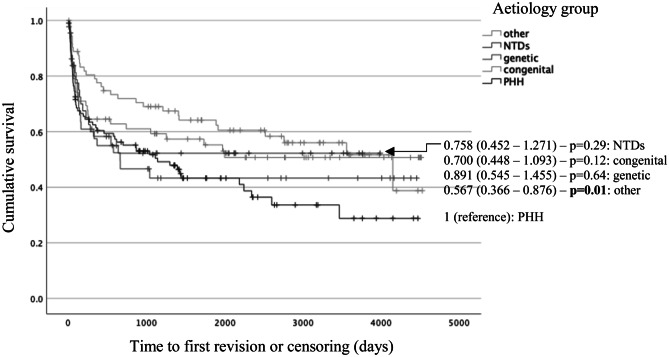
Univariate survival function for VP shunt by aetiology group. The hazard of overall shunt revision in each aetiology group relative to PHH can be found next to the relevant Kaplan Meier curve, alongside the confidence intervals and *p* value. Poorest shunt survival was observed in PHH; the “other,” congenital, NTD, and genetic groups had hazards of shunt revision which were reduced by 43.3% (*p* = 0.01), 30.0% (*p* = 0.12), 24.2% (*p* = 0.29), and 10.9% (*p* = 0.64), respectively, relative to PHH. *PHH*, post-haemorrhagic hydrocephalus; *NTDs*, neural tube defects; *VP*, ventriculoperitoneal

In a univariate analysis of time to shunt failure, PHH had the poorest shunt survival, with all other groups having lower hazard ratios; only the “other” group was significantly different (Fig. 1). In a multivariate analysis, gestational age at birth and corrected age at VP shunt insertion were the significant factors affecting shunt survival; when other predictors were kept constant, every week’s increase in gestational age at birth decreased the hazard of shunt revision by 6.9% (*p* = 0.01), and every week’s increase in corrected age at shunt insertion decreased the hazard of revision by 2.0% (*p* = 0.01). Sex, birthweight, and type of shunt valve were not included in the multivariate model, as they did not have a significant effect on hazard of shunt revision. A higher univariate hazard of shunt revision was seen in preterm PHH compared to term IVH (Supplementary Information 2).

#### Shunt complications

The most common reason for shunt revision was obstruction (61.3%), followed by infection (14.4%), migration (12.5%), CSF leak (7.7%), and over-drainage (1.9%). Reasons for revision did not differ significantly between aetiologies. Beyond the first year post-shunt insertion, revisions due to shunt infection significantly dropped by 8.4% (*p* = 0.04), and those involving obstruction rose by 15.9% (*p* < 0.01). Aetiology had no significant effect on the region of obstruction, with most obstructions involving the proximal catheter (70.3%).

### Neurodevelopmental outcome

**Fig. 2:**
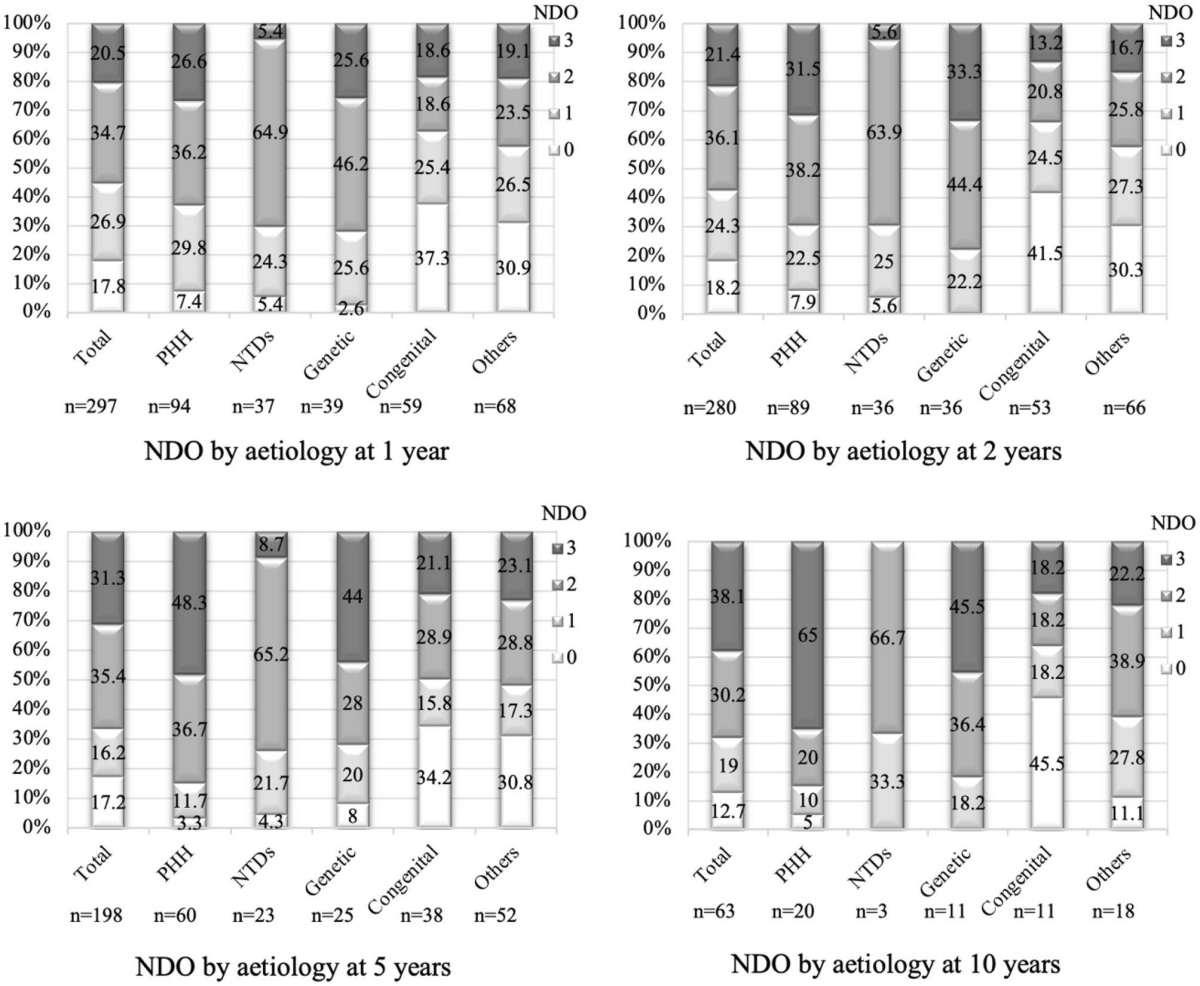
Distribution of NDO scores by aetiology at 1, 2, 5, and 10 years. The PHH and genetic hydrocephalus groups had the highest percentage of “3 s” (severe disability) at all four time points. The NTD group scored significantly more “2 s” (disability) and significantly fewer “3 s” than PHH at every time (*p* < 0.01) except 10 years, likely due to small sample size (*n* = 3 NTDs). The congenital and “other” hydrocephalus groups had significantly more “0 s” (normal outcome) than PHH at every time point (except 10 years for “other”) (*p* < 0.01), and significantly fewer “3 s” than PHH at 5 and 10 years (*p* < 0.01). *PHH*, post-haemorrhagic hydrocephalus; *NTDs*, neural tube defects; *NDO*, neurodevelopmental outcome

There were significant overall differences in NDO between aetiologies at 1 (*p* < 0.05), 2 (*p* = 0.01), and 5 (*p* < 0.01) years, while 10-year differences did not reach significance due to small sample size (*p* = 0.14) (Fig. 2). At all four time points, the largest percentages of infants scoring a “3” (severe disability) occurred in PHH and genetic hydrocephalus, increasing sequentially from 26.6% and 25.6%, respectively, at 1 year, to 31.5% and 33.3% (2 years), 48.3% and 44.0% (5 years), and 65.0% and 45.5% (10 years).

In a multivariate ordinal logistic regression model (Table 5), there were significant differences in NDO between aetiologies at all time points, apart from 10 years. The odds of a poorer NDO, after controlling for gestational age at birth, corrected age at shunt insertion, shunt infections, and number of revisions up to that time point, remained significantly lower in congenital and “other” hydrocephalus than in PHH at every time point, except 1 and 10 years when the “other” group was no longer significantly different to PHH (*p* = 0.11, *p* = 0.28, respectively). Outcomes compared to PHH were not significantly different in the NTD and genetic groups; additionally, none of the a priori predictors had a significant effect on NDO. A direct comparison between term IVH and preterm PHH demonstrated significantly higher odds of a poorer NDO in preterm PHH at 5 (*p* = 0.01) and 10 years (*p* = 0.04) (Supplementary Information 2).

**Table 5:** NDO multivariate regression results

Time point	Odds ratio (95% confidence intervals)					
	PHH	NTDs	Genetic	Congenital	Other	*p* value
NDO at 1 year	*n* = *93* 1 (reference)	*n*=*37* 0.811 (0.291–2.261)	*n*=*38* 1.382 (0.500–3.819)	*n*=*57* 0.289 (0.107–0.780)*	*n*=*68* 0.429 (0.152–1.207)	0.01
NDO at 2 years	*n* = *89* 1 (reference)	*n*=*35* 0.577 (0.197–1.691)	*n*=*34* 1.353 (0.459–3.990)	*n*=*53* 0.186 (0.065–0.533)*	*n*=*65* 0.299 (0.102–0.881)*	<0.01
NDO at 5 years	*n* = *60* 1 (reference)	*n*=*23* 0.300 (0.078–1.158)	*n*=*25* 0.660 (0.176–2.470)	*n*=*38* 0.187 (0.050–0.708)*	*n*=*51* 0.201 (0.053–0.759)*	0.03
NDO at 10 years	*n* = *20* 1 (reference)	*n*=*3* 0.205 (0.016–2.645)	*n*=*11* 0.825 (0.107–6.370)	*n*=*11* 0.087 (0.009–0.831)*	*n*=*18* 0.305 (0.035–2.674)	0.09

Sex, birthweight, type of shunt valve, and presence or absence of shunt revision by that time point had no significant effect on NDO after adjusting for other predictors so they were excluded from the final multivariate model

*PHH* post-haemorrhagic hydrocephalus, *NTDs* neural tube defects, *NDO* neurodevelopmental outcome

^*^Parameters that are statistically significant between that aetiology group and PHH (using ordinal logistic regression)

### Other clinical outcomes

In separate multivariate analyses which controlled for different predictors, including gestational age, corrected age at shunt insertion, and shunt revisions (Table 6), significant differences were observed between PHH and other aetiologies in odds of CP (*p* = 0.01), epilepsy (*p* < 0.01), speech delay (*p* < 0.01), and mortality (*p* = 0.01). CP rates were highest in PHH (62.6%), while epilepsy rates were highest in genetic hydrocephalus (55.2%). In general, the odds of most comorbidities were lower for all aetiology groups compared to PHH, except for genetic hydrocephalus which had significantly higher odds of endocrine dysfunction (*p* = 0.01) and mortality (*p* = 0.04). The effects of other predictors in the multivariate models are displayed in Table 6; importantly, each revision < 2 years was significantly associated with increased odds of CP by 34.1% (*p* = 0.04).

**Table 6:** Univariate (U) or multivariate (M) regression results for clinical outcomes by hydrocephalus aetiology

Outcome	Odds ratio (95% confidence intervals)/median (range)					
	PHH	NTDs	Genetic	Congenital	Other	*p* value
CP (M)	*n* =* 85* 1 (reference)	*n* = *34* 0.022 (0.002–0.206)*	*n* = *26* 0.310 (0.083–1.158)	*n* = *38* 0.221 (0.059–0.820) *	*n* = *54* 0.338 (0.101–1.128)	0.01
Epilepsy (M)	*n* = *94* 1 (reference)	*n* = *36* 0.133 (0.030–0.581)*	*n* = *29* 1.320 (0.384–4.537)	*n* = *54* 0.630 (0.199–1.993)	*n* = *67* 0.383 (1.114–1.281)	< 0.01
Speech delay (M)	*n* = *93* 1 (reference)	*n* = *29* 0.648 (0.234–1.792)	*n* = *38* 4.230 (0.914–19.572)	*n* = *52* 0.272 (0.125–0.592)*	*n* = *63* 0.240 (0.114–0.502)*	< 0.01
Schooling (U)	*Mainstream school with support *(reference: mainstream school)					
	*n* = *50* 1 (reference)	*n* = *23* 1.556 (0.447–5.413)	*n* = *25* 2.000 (0.334–11.969)	*n* = *38* 0.407 (0.136–1.220)	*n* = *50* 0.293 (0.101–0.854)*	< 0.01
	*Special school *(reference: mainstream school)					
	*n* = *50* 1 (reference)	*n* = *23* 0.200 (0.042–0.959)*	*n* = *25* 3.400 (0.661–17.500)	*n* = *38* 0.200 (0.068–0.592)*	*n* = *50* 0.224 (0.084–0.598)*	< 0.01
Behavioural disorders (M)	*n* = *88* 1 (reference)	*n* = *35* 0.871 (0.220–3.449)	*n* = *29* 1.572 (0.416–5.939)	*n* = *53* 2.601 (0.799–8.468)	*n* = *64* 2.047 (0.594–7.051)	0.23
Endocrine dysfunction (U)	*n* = *87* 1 (reference)	*n* = *35* 0.693 (0.137–3.510)	*n* = *34* 4.114 (1.390–12.176)*	*n* = *53* 1.190 (0.358–3.961)	*n* = *64* 1.404 (0.467–4.222)	0.06
Mortality (M)	*n* = *96* 1 (reference)	*n* = *35*	*n* = *41* 6.070 (1.137–32.390)*	*n* = *60* 1.771 (0.306–10.248)	*n* = *70* 0.932 (0.131–6.630)	0.01
Age at death (years)˟	*n = 7* 1.91 (0.70–5.18)	*n* = *0*	*n* = *12* 1.16 (0.02–5.62)	*n* = *7* 1.42 (0.81–5.63)	*n* = *4* 1.93 (0.31–4.69)	0.69

*Predictors included in the final model varied by clinical outcome are as follows:*

CP: birthweight (a priori — gestational age excluded due to high multicollinearity) and number of revisions < 2 years of age (significant in model: significantly increased the odds of CP by 34.1% for every revision, *p* = 0.04)

Epilepsy: gestational age (a priori), corrected age at VP shunt insertion (a priori), shunt revision ≤ 1 year of age (a priori), number of revisions < 1 year of age (a priori), and shunt infection ≤ 1 year of age (a priori)

Speech delay: sex (significant in model: male sex significantly increased odds of speech delay by 49.5%, *p* = 0.02)

Schooling: there is no multivariate model for schooling as it was not significantly affected by any of the possible predictors included in model building, nor were any predictors indicated for a priori inclusion

Mortality: gestational age (a priori) and corrected age at VP shunt insertion (a priori and significant in model: odds of mortality decreased by 0.6% for every week older the infant was at shunt insertion, *p* = 0.04)

Behavioural outcome: gestational age (a priori), number of revisions before age 2 (a priori), and sex (a priori and significant in model: male sex significantly increased odds of behavioural disorders by 43.3%, *p* < 0.05)

Endocrine dysfunction: there is no multivariate model as the outcome was not significantly affected by any of the possible predictors, nor were any predictors indicated for a priori inclusion

*PHH* post-haemorrhagic hydrocephalus, *NTDs* neural tube defects, *CP* cerebral palsy

˟Median (range)

^*^Parameters that are *statistically significant* between that aetiology group and PHH (using logistic regression)

In terms of likelihood of attending special school, a univariate analysis demonstrated significantly higher odds in PHH than in “other” (*p* < 0.01), congenital (*p* < 0.01), and NTD-associated hydrocephalus (*p* = 0.04), with the highest proportion of children attending special school in genetic (68.0%) and PHH (50.0%) and the highest attending mainstream school with support in NTDs (60.9%). Behavioural disorders did not differ significantly by aetiology (*p* = 0.23), nor did types of behavioural disorders; overall, the commonest disorders were autism and social communication difficulties (13.0%), sleep difficulties (11.5%), and challenging behaviours (10.7%).

The commonest causes of endocrine dysfunction were adrenocortical insufficiency (3.4%), growth hormone deficiency (GHD) (3.3%), hypothyroidism (2.2%), and central precocious puberty (2.2%); only GHD showed significant differences between PHH (0.0%) and genetic hydrocephalus (14.7%, *p* < 0.01). Median age at death was not significantly different between aetiology groups (1.41 years, *p* = 0.69). Only one death was shunt-related due to suspected shunt infection in a patient with congenital hydrocephalus.

### Sensitivity analysis

The sensitivity analysis conducted to separate the effects of concomitant pathology on NDO, and other clinical outcomes resulted in largely unchanged conclusions (Supplementary Information 3).

## Discussion

PHH comprises a significant proportion of infant hydrocephalus, and providing optimal surgical management for these infants, with the objective of improving surgical and developmental outcome, is crucial. The objective of this retrospective cohort study was to present an overall contemporary picture of the burden of care, both surgical and neurodevelopmental, related to infant hydrocephalus in a cohort managed in a single institution, and to underline the particular impact of PHH compared to other causes of hydrocephalus in infancy. The results demonstrated that PHH has the poorest univariate shunt survival and the highest rate of severe disability.

### Surgical outcome

Shunt failures are common in paediatric hydrocephalus and are associated with significant morbidity [7]. In this study, shunt survival was poorest in the PHH group, with revision rates and number of revisions beyond a year and within 5 years significantly higher in PHH than in congenital or “other” hydrocephalus. This is consistent with Paulsen et al. who demonstrated more frequent revisions in the PHH group than other aetiologies, and Notarianni et al. who similarly showed higher 5-year shunt failure rates in PHH compared to congenital and “other” hydrocephalus, with no significant differences between PHH and myelomeningoceles [6, 20].

Higher shunt revision rates in the PHH group may be explained by the relatively immature immune systems, increased CSF protein, variable inflammatory responses to CSF blood, small abdominal cavity, and thin skin, which is prone to breakdown [16, 21]. Other studies have, however, shown conflicting findings; post-infectious hydrocephalus, congenital hydrocephalus, tumours, trauma, and myelomeningoceles have all been associated with the highest shunt failure rates in comparable series [3, 7, 22–25]. This may be due to the absence of a PHH group in some studies, differing patient demographics and inclusion criteria, or small numbers for some aetiologies in the present study, particularly tumours, trauma, and post-infectious hydrocephalus [22].

In a multivariate survival analysis, both gestational age and corrected age at shunt insertion were independent risk factors for shunt failure, in agreement with the literature [7, 26, 27]. This reaffirms that prematurity-related factors likely contribute to the poor shunt survival seen in PHH. This series showed a reduction in the risk of shunt revision by 6.9% for every weekʼs increase in gestational age and a reduction of 2.0% for every weekʼs increase in corrected age at shunt insertion. This suggests that shunt insertion in neonates should be deferred if at all possible. PHH infants have been shown to have an increased risk of multiple shunt failures as they age, suggesting that additional factors such as shunt infection may predispose them to multiple shunt failures [28, 29].

While not statistically significant, PHH had the highest infection rate (17.9% of revisions), likely due to remnants of haemorrhagic material and poor immunity [21, 23]. While other studies have also demonstrated high infection rates in post-infectious hydrocephalus, this subgroup was too small within the present study to draw similar conclusions [30]. Infections dropped in frequency after the first year of shunt insertion, in agreement with Simon et al.’s finding that most CSF shunt infections occur within the first 12 months of shunt placement [31].

### Neurodevelopmental outcome

Severe neurological disability was most prevalent in PHH and genetic hydrocephalus, while children with NTDs had more mild disabilities in relation to the spinal lesion; children in the congenital and “other” hydrocephalus had the most normal outcomes. This is consistent with previous reports that cognitive impairment, speech and language difficulties, and seizures were most common in PHH, while musculoskeletal problems predominated in myelomeningoceles [32]. In PHH and genetic hydrocephalus, the percentage of severe disabilities increased with time, up to 65.0% and 45.5%, respectively, at 10 years. This time-dependent trend has been described and underlines the long-term nature of the care that these children and young people require [33].

Poor outcomes in PHH may be due to prematurity, ischaemic, or inflammatory insults to periventricular brain structures, subsequent white matter injury, or increased shunt complications [32, 34, 35]. Prematurity alone is associated with motor and cognitive disability [12]; however, this is significantly higher in those infants who also have severe IVH and consequent PHH. A large study showed that in children born under 28 weeks’ gestation and assessed at 8 years of age, only 12% had impaired intellectual ability; in addition, only 24% had motor dysfunction [36]. Our cohort only selects premature neonates who also had PHH, with consequently poorer outcomes. Genetic hydrocephalus has also been associated with neural cell fate alterations in the cerebral cortex, suggesting a shared pathogenic process with PHH, potentially accounting for the similar outcomes of both aetiologies [37].

### Other clinical outcomes

Several studies have measured cognition in hydrocephalic children using intelligence quotient [38, 39]. Hirsch, however, suggested that the best guide of functional outcome is attendance of mainstream school [40]. In this series, the proportion of children attending special school was highest in genetic and PHH, supporting the NDO results indicating poorest outcomes in these aetiologies. The study also demonstrated that NTDs had the highest percentage of children attending mainstream school with support but significantly the lowest attending special school (13.0%), consistent with previous findings that infants with myelomeningoceles have, and maintain, improved cognition [39].

This series also observed that the highest CP rates were in the PHH group (62.6%), likely due to diffuse post-haemorrhagic brain injury and parenchymal infarctions [41]. The number of shunt revisions was independently associated with CP and has been previously shown to correlate with number of neurological impairments [2]. As expected, epilepsy risk was highest in genetic hydrocephalus (55.2%) due to associated cortical malformations, but significantly lower in NTDs (11.1%) than in PHH (43.2%) [42]. Odds of speech delay were highest in genetic hydrocephalus, and only congenital and “other” hydrocephalus had significantly lower odds of speech impairment than PHH, as seen in previous literature [32]. The behavioural disorder rate for hydrocephalic children in this study was 31.1%, similar to the 33% teacher-reported behavioural difficulties rate obtained in studies which used standardised questionnaires [43].

The commonest endocrine disorders overall were adrenocortical insufficiency, GHD, hypothyroidism, and central precocious puberty; these are all associated with anterior pituitary dysfunction, supporting the hypothesis that ventricular dilation or raised ICP may disturb the hypothalamic-pituitary axis, resulting in hypopituitarism [44]. Odds of endocrine dysfunction were significantly higher in genetic hydrocephalus than in PHH; some congenital conditions associated with hydrocephalus, such as midline malformations, also independently affect the hypothalamo-pituitary axis [45]. Finally, mortality was highest in genetic hydrocephalus and lowest in NTDs, with a PHH mortality rate of 7.3%; this is lower than the rates of 46% reported in the literature due to differing study follow-up times and, potentially, improvements in neonatal care over time [46].

### Limitations

As this was a retrospective study, it was not possible to evaluate children using the Bayley Scales of Infant Development [47]. After consideration of multiple developmental scales, the scale developed by Resch et al. and utilised by van Zanten et al. was deemed the most comprehensive and reliable due to utilisation of the greatest combination of developmental, motor, and sensory sequelae of hydrocephalus (Table 1) [18, 48–52]. There was sufficient information from multiple sources to reliably assign outcome scores for patients, allowing the collection of NDO data for a large population over 10 years. However, while all evaluations of NDO were undertaken by the same operator (MM) to ensure consistency, the lack of an independent check by another reviewer is a limitation of this study. Loss to follow-up, death, missing data, and failure to reach the age for specific assessments also meant that patient numbers in certain analyses were smaller than in the original sample.

Although all the patients were treated in the same institution, there was a modification to the management algorithm for PHH from 2013, which included an effort to encourage earlier referral of these neonates with earlier implantation of TNPs. Timing of the first intervention in PHH is still controversial, although some evidence is emerging that early treatment may be beneficial [53, 54]. From 2018, a small number of neonates also underwent neuroendoscopic lavage at the time of VSGS insertion. The protocol for definitive VP shunting following PHH, however, remained the same throughout the study, with infants with persisting hydrocephalus undergoing insertion of a VP shunt at term-equivalent age and/or when their weight had reached 2 kg.

As the study was non-randomised, unidentified confounders may have biased the results; however, a sensitivity analysis controlling for the effects of concomitant pathology on outcomes demonstrated largely unchanged conclusions. For myelomeningoceles, the effects of the spinal lesion on NDO could not be differentiated from the effects of the hydrocephalus, although previous research observed more frequent motor impairments in myelomeningoceles with hydrocephalus than those without [55]. Other clinical outcomes, such as CP, speech delay, and behavioural disorders, were evaluated clinically without using validated prospective scales that account for severity. Furthermore, over the long study course, the treating neurosurgeons, neonatologists, and clinical practices may have varied within the cohort.

## Conclusion

This study provides a contemporaneous picture of the burden of care related to infant hydrocephalus, as evaluated in our unit over the last 10 years. Surgical outcomes and NDOs are worst for PHH and genetic causes of hydrocephalus. In most patients, disability worsens with age, underlining the importance of long-term neurodevelopmental and neurosurgical support and review. Further research to reduce the incidence and severity of IVH in premature neonates and to minimise their subsequent cognitive impairment in the context of PHH is urgently needed.

## Supplementary information

Below is the link to the electronic supplementary material.

Supplementary file1 (DOCX 14 KB)

Supplementary file2 (DOCX 24 KB)

Supplementary file3 (DOCX 44 KB)

## Data Availability

The datasets generated during the current study are available from the corresponding author on reasonable request.
