# Supplementary material for: Post-haemorrhagic hydrocephalus is associated with poorer surgical and neurodevelopmental sequelae than other causes of infant hydrocephalus
Source: Childs Nerv Syst. 2021 Jun 19;37(11):3385–96. doi: 10.1007/s00381-021-05226-4 (PMC8578110; doi:10.1007/s00381-021-05226-4)
Supplement: Supplementary file 1 — Supplementary file1 (DOCX 14 KB) [file 381_2021_5226_MOESM1_ESM.docx]

# **Post-haemorrhagic hydrocephalus is associated with poorer surgical and neurodevelopmental sequelae than other causes of infant hydrocephalus**

Child’s Nervous System

# Malak Mohamed, Saniya Mediratta, Aswin Chari, Cristine Sortica da Costa, Greg James, William Dawes, Kristian Aquilina

**Corresponding Author:**

Malak Mohamed

E-mail: [malakmohamedz@hotmail.com](mailto:malakmohamedz@hotmail.com)

UCL Great Ormond Street Institute of Child Health, University College London, London, United Kingdom

Leeds School of Medicine, University of Leeds, Leeds, United Kingdom

### Supplementary information 3: sensitivity analysis excluding infants with concomitant pathology

Whilst it is recognised that underlying aetiology is a predictor of outcome, in certain cases such as brain tumours, it was difficult to determine whether resulting visual impairments were due to the compressive effects of the tumour on the optic pathway, or due to the hydrocephalus. Similarly, hearing loss in postinfectious hydrocephalus could have been due to meningitis rather than the resulting hydrocephalus, and poor outcome in posttraumatic hydrocephalus may have been due to traumatic brain injury as opposed to hydrocephalus. For cases where outcomes were highly likely to be due to concomitant pathology as opposed to hydrocephalus, a sensitivity analysis was performed which excluded these infants, to investigate whether conclusions changed significantly.

The sensitivity analysis demonstrated similar conclusions for one-year NDO, although in the multivariate analysis, congenital hydrocephalus no longer had significantly lower odds of poorer NDO than PHH (p=0.10). At two and five years, congenital and ‘other’ hydrocephalus remained with significantly lower odds of poorer NDO than PHH, but in the multivariate analysis, the ‘other’ group lost its significance at two years (p=0.06) and the congenital hydrocephalus group at five years (p=0.08). Ten-year NDO conclusions were largely unchanged, although in the multivariate analysis, congenital hydrocephalus was no longer significantly different from PHH (p=0.47). It is likely that the reduced sample size in the sensitivity analysis is responsible for these losses of significance, as conclusions have otherwise remained unchanged.

Conclusions regarding the effects and significance of aetiology on odds of CP, speech delay, schooling and behavioural problems were also largely unchanged, although in the multivariate analysis for behavioural disorders, congenital hydrocephalus had significantly higher odds of behavioural problems than PHH (odds ratio (OR) 3.939 (1.039–14.931), p=0.04). Conclusions for epilepsy and endocrine dysfunction were also similar, although the higher odds of epilepsy in PHH compared to ‘other’ hydrocephalus became statistically significant (OR 0.442 (0.203–0.965), p=0.04), while odds of endocrine dysfunction in genetic conditions were no longer significantly different to PHH (OR 2.219 (0.501–9.828), p=0.29). Finally, in terms of mortality, genetic hydrocephalus no longer had significantly worse odds of mortality than PHH (OR 0.768 (0.054–11.013), p=0.85).
