# Supplementary material for: Post-haemorrhagic hydrocephalus is associated with poorer surgical and neurodevelopmental sequelae than other causes of infant hydrocephalus
Source: Childs Nerv Syst. 2021 Jun 19;37(11):3385–96. doi: 10.1007/s00381-021-05226-4 (PMC8578110; doi:10.1007/s00381-021-05226-4)
Supplement: Supplementary file 2 — Supplementary file2 (DOCX 24 KB) [file 381_2021_5226_MOESM2_ESM.docx]

# **Post-haemorrhagic hydrocephalus is associated with poorer surgical and neurodevelopmental sequelae than other causes of infant hydrocephalus**

Child’s Nervous System

# Malak Mohamed, Saniya Mediratta, Aswin Chari, Cristine Sortica da Costa, Greg James, William Dawes, Kristian Aquilina

**Corresponding Author:**

Malak Mohamed

E-mail: [malakmohamedz@hotmail.com](mailto:malakmohamedz@hotmail.com)

UCL Great Ormond Street Institute of Child Health, University College London, London, United Kingdom

Leeds School of Medicine, University of Leeds, Leeds, United Kingdom

### Supplementary information 2: comparison in outcomes between preterm PHH and term IVH

In our study, term IVH was treated as a separate entity to PHH of prematurity. The pathophysiology of the brain injury in these neonates is different from that in preterm IVH; there is no germinal matrix injury, no periventricular infarction, and brain development is at a later stage. Management was also different; if there was acute hydrocephalus, infants who suffered term IVH underwent external ventricular drainage rather than insertion of a VSGS or an access device. When necessary, they underwent VP shunt insertion as soon as the intraventricular blood resolved.

Nonetheless, a direct comparison between term IVH and preterm PHH was performed, to observe for differences in outcomes between these groups, based on prematurity. Revision rates were higher in preterm PHH than in term IVH, although this did not reach significance (Table 7). The univariate hazard of shunt revision in term IVH was 0.591 (0.317 – 1.101) compared to preterm PHH (p=0.10).

| **Table 7: Surgical outcomes in preterm PHH compared to term IVH** | | | |
| --- | --- | --- | --- |
| **Parameter** **Frequency (%)/median (range)** | | | |
|  | **Preterm PHH** | **Term IVH** | **p-value** |
| **Excluding those with follow-up <1 year of shunt insertion:** | | | |
|  | **n=96** | **n=28** |  |
| **Revision rate <1 year** | 36 (37.5%) | 7 (25.0%) | 0.22 |
| **Number of revisions** <**1 year of shunt** | 0 (0 – 4) | 0 (0 – 3) | 0.23 |
| **Excluding those with follow-up <5 years of shunt insertion:** | | | |
|  | **n=59** | **n=21** |  |
| **Revision rate >1 year** | 24 (40.7%) | 4 (19.0%) | 0.07 |
| **Number of revisions >1 year of shunt** | 0 (0 – 5) | 0 (0 – 3) | 0.16 |
| **5-year revision rate** | 37 (62.7%) | 9 (42.9%) | 0.11 |
| **Total number of revisions <5yrs per patient** | 1 (0 – 9) | 0 (0 – 3) | 0.11 |
| As each patient had their shunt in-situ for different lengths of time, evaluation of revisions beyond a year of insertion was limited to patients with a minimum of five years of follow-up post-shunt, and only five-year follow-up was evaluated.  **Revision rates** are defined as the proportion of patients who required VP shunt revisions due to shunt complications.  P-values were obtained using:  **Chi-squared test:** revision rate <1 year, revision rate >1 year, 5-year revision rate  **Kruskall-Wallis test:** number of revisions <1 year of shunt, number of revisions >1 year of shunt, total number of revisions <5yrs per patient  PHH = post-haemorrhagic hydrocephalus; IVH = intraventricular haemorrhage; VP = ventriculoperitoneal | | | |

As for NDO, significantly higher odds of a poorer NDO were observed in preterm PHH compared to term IVH at five (p=0.01) and ten years (p=0.04) in a multivariate analysis, controlling for gestational age at birth, corrected age at shunt insertion, shunt infections and number of revisions up to that time point (Table 8).

| **Table 8: NDO multivariate regression results for preterm PHH versus term IVH** | | | |
| --- | --- | --- | --- |
| **Time point Odds ratio (95% confidence intervals)** | | | |
|  | **Preterm PHH** | **Term IVH** | **p-value** |
| **NDO at 1 year** | **n = 93**  1 (reference) | **n=27**  0.251 (0.053 – 1.181) | 0.08 |
| **NDO at 2 years** | **n=89**  1 (reference) | **n=25**  0.269 (0.053 – 1.371) | 0.11 |
| **NDO at 5 years** | **n = 60**  1 (reference) | **n=19**  0.052 (0.005 – 0.517) | **0.01** |
| **NDO at 10 years** | **n = 20**  1 (reference) | **n=5**  0.010 (0.000 – 0.721) | **0.04** |
| Sex, birthweight, type of shunt valve and presence or absence of shunt revision by that time point had no significant effect on NDO after adjusting for other predictors so were excluded from the final multivariate model.  PHH = post-haemorrhagic hydrocephalus; IVH = intraventricular haemorrhage; NDO = neurodevelopmental outcome | | | |

While the odds of CP, epilepsy, speech delay, special schooling, and mainstream schooling with support were higher in preterm PHH, they were only significant in speech delay (p=0.02) and mainstream schooling with support (p=0.04). Odds of behavioural disorders were significantly higher in term IVH (p=0.03).

| **Table 9: Univariate (U) or multivariate (M) regression results for clinical outcomes in preterm PHH versus term IVH** | | | |
| --- | --- | --- | --- |
| **Outcome** **Odds ratio (95% confidence intervals)** | | | |
|  | **Preterm PHH** | **Term IVH** | **p-value** |
| **CP**  **M** | **n=85**  1 (reference) | **n=26**  0.459 (0.072 – 2.913) | 0.41 |
| **Epilepsy**  **M** | **n=94**  1 (reference) | **n=27**  0.289 (0.051 – 1.624) | 0.16 |
| **Speech delay**  **M** | **n = 93**  1 (reference) | **n=26**  0.332 (0.127 – 0.864) | **0.02** |
| **Schooling**  **U**    **U** | **Mainstream school with support** (reference: mainstream school) | | |
|  | **n=50**  1 (reference) | **n=21**  0.200 (0.044 – 0.913) | **0.04** |
|  | **Special school** (reference: mainstream school) | | |
|  | **n=50**  1 (reference) | **n=21**  0.320 (0.098 – 1.046) | 0.06 |
| **Behavioural disorders**  **M** | **n=88**  1 (reference) | **n=27**  8.703 (1.182 – 64.083) | **0.03** |
| **Endocrine dysfunction**  **U** | **n=87**  1 (reference) | **n=27**  1.429 (0.343 – 5.954) | 0.62 |
| **Mortality**  **M** | **n=96**  1 (reference) | **n=27**  There were no deaths in this group | 1.00 |
| Predictors included in the final model varied by clinical outcome:  **CP:** birthweight (a priori – gestational age excluded due to high multicollinearity); number of revisions <2 years of age (significant in model: significantly increased the odds of CP by 34.1% for every revision, p=0.04)  **Epilepsy:** gestational age (a priori), corrected age at VP shunt insertion (a priori), shunt revision <1 year of age (a priori), number of revisions <1 year of age (a priori), shunt infection <1 year of age (a priori)  **Speech delay:** sex (significant in model: male sex significantly increased odds of speech delay by 49.5%, p=0.02)  **Schooling:** there is no multivariate model for schooling as it was not significantly affected by any of the possible predictors included in model building, nor were any predictors indicated for a priori inclusion.  **Mortality:** gestational age (a priori) and corrected age at VP shunt insertion (a priori and significant in model: odds of mortality decreased by 0.6% for every week older the infant was at shunt insertion, p=0.04)  **Behavioural outcome:** gestational age (a priori), number of revisions before age 2 (a priori) and sex (a priori and significant in model: male sex significantly increased odds of behavioural disorders by 43.3%, p<0.05)  **Endocrine dysfunction:** there is no multivariate model as the outcome was not significantly affected by any of the possible predictors, nor were any predictors indicated for a priori inclusion.  PHH = post-haemorrhagic hydrocephalus; IVH = intraventricular haemorrhage; CP = cerebral palsy | | | |
